# Supplementary material for: 3D quantification of nanolites using X-ray ptychography reveals syn-eruptive nanocrystallisation impacts magma rheology
Source: Nat Commun. 2025 Aug 1;16:7083. doi: 10.1038/s41467-025-62444-z (PMC12317098; doi:10.1038/s41467-025-62444-z)
Supplement: Supplementary file 1 — Supplementary Information [file 41467_2025_62444_MOESM1_ESM.pdf]

3D quantification of nanolites using X-ray ptychography reveals syn-eruptive nanocrystallisation impacts magma rheology

Emily C. Bamber [a,b*], Fabio Arzilli [c], Silvia Cipiccia [d,e], Darren J. Batey [e], Giuseppe La Spina [f], Margherita Polacci [b], Ali Gholinia [g], Heath Bagshaw [h], Danilo Di Genova [a], Richard Brooker [i], Daniele Giordano [j], Pedro Valdivia [a,k] and Mike R. Burton [b]

[a] Institute of Science, Technology and Sustainability for Ceramics (ISSMC), National Research Council (CNR), Via Granarolo 64, 48018, Faenza, Italy

[b] Department of Earth and Environmental Sciences, The University of Manchester, Oxford Road, Manchester, M13 9PL, UK

[c] School of Science and Technology, Geology Division, University of Camerino, Camerino, Italy

[d] Department of Medical Physics and Biomedical Engineering, University College London, London, WC1E 6BT, UK

[e] Diamond Light Source, Harwell Science and Innovation Campus, Fermi Avenue, Didcot, OX11 0DE, UK

[f] Istituto Nazionale di Geofisica e Vulcanologia - Osservatorio Etneo, Sezione di Catania, Piazza Roma, 2, 95125, Catania, Italy

[g] Department of Materials, The University of Manchester, Oxford Road, Manchester, M13 9PL, UK

[h] School of Engineering, The University of Liverpool, Liverpool, L69 3GQ, UK

[i] School of Earth Sciences, University of Bristol, Bristol, BS8 1RL, UK

[j] Department of Earth Sciences, University of Turin, Via Valperga Caluso, 35, 10125, Turin, Italy

[k] Bayerisches Geoinstitut, University of Bayreuth, Universitätsstraße 30, 95440, Bayreuth, Germany

Supplementary Figures and Tables

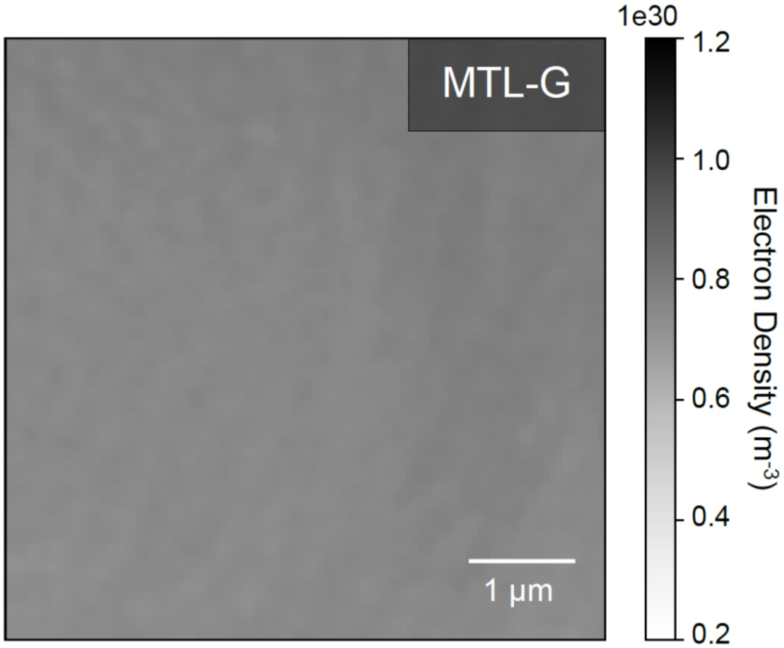

Supplementary Figure 1: An orthoslice of homogeneous basaltic glass sample MTL-G, obtained using X-ray ptychography.

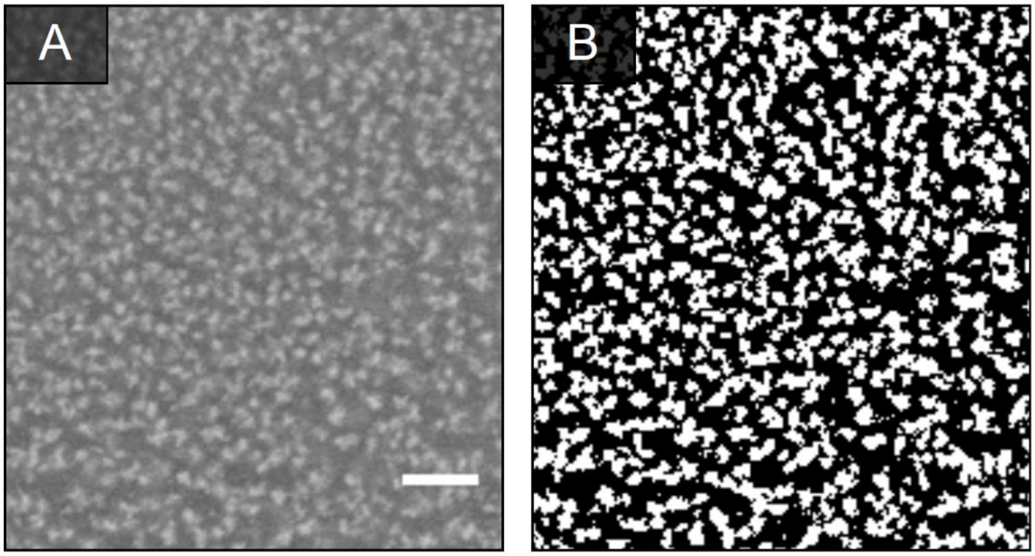

Supplementary Figure 2: (a) A back-scattered electron (BSE) image of nanolites in a clast of the Fontana Lapilli eruption. The image is an area of Fig. 2d at higher magnification. Also shown in **(b)** is the nanolite phase, where the segmentation was performed using ImageJ[1]. The scale bar is equal to 2 μm.

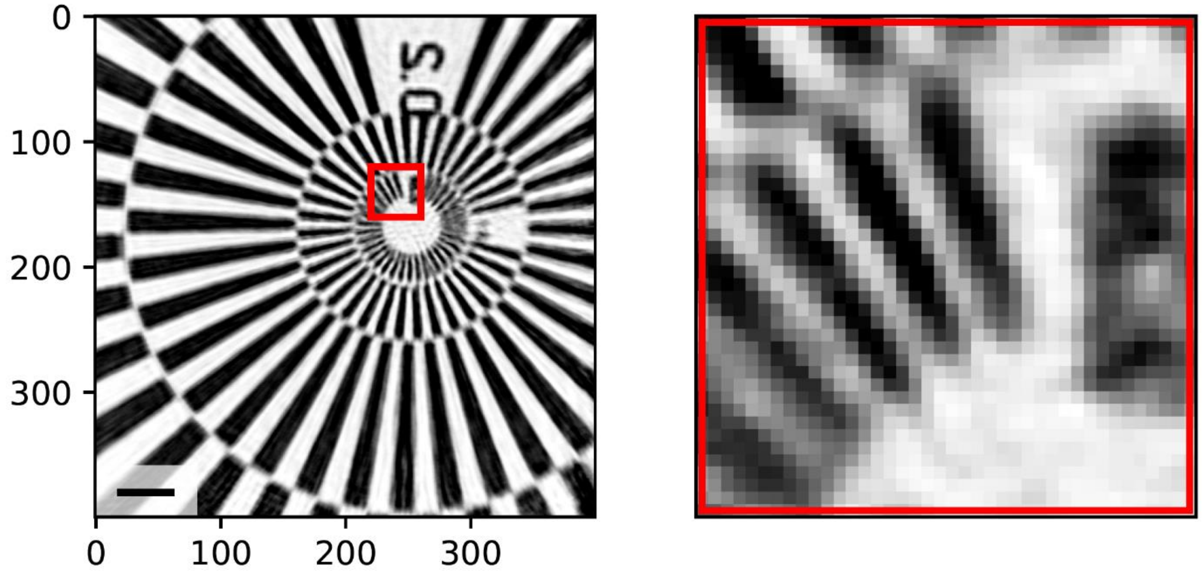

Supplementary Figure 3: Siemens star test pattern imaged at the I13-1 beamline during the X-ray ptychography experiment. Reconstructed pixel size, 28 nm. The scale bar represents 1 μm.

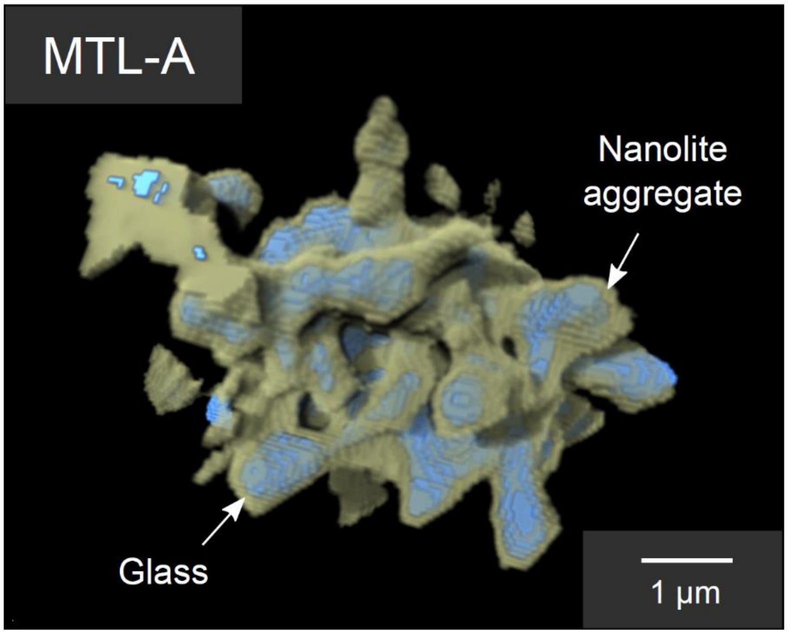

Supplementary Figure 4: Volume rendering of the aggregate observed in sample MTL-A, showing the evolved, Fe-depleted glass (highlighted in yellow) which surrounds the aggregate and the isolated nanolites. The volume rendering was produced using VGStudio.

	**Bulk composition** *Crystal-free basalt*	**Evolved glass** *Trachy-andesite*
**SiO_2**	51.81	58.17
**TiO_2**	1.44	1.27
**Al_2O_3**	14.39	14.26
**FeO**	13.67	10.63
**MnO**	0.27	0.26
**MgO**	4.86	2.24
**CaO**	8.75	5.71
**Na_2O**	2.94	3.88
**K_2O**	1.48	3.03
**P_2O_5**	0.35	0.54
**Cr_2O_3**	0.04	0
**Total**	100	100

Supplementary Table 1: The melt compositions used in the viscosity model calculations, representing the crystal-free MTL melt inclusion composition[2] and the evolved, trachy-andesitic FL composition of the matrix glass following nanolite crystallisation[3]. Both compositions are normalised to anhydrous compositions and represent an average, acquired using EPMA.

Label	Long axis (nm)	Short axis (nm)	Aspect ratio	Volume (nm^3)	Type
1	6332	3995	1.58	1.05×10^{10}	Aggregate
2	2042	520	3.93	2.01×10^{8}	Aggregate
3	1586	747	2.12	2.40×10^{8}	Aggregate
4	1391	597	2.33	1.76×10^{8}	Aggregate
5	1388	716	1.94	2.45×10^{8}	Aggregate
6	905	379	2.39	6.85×10^{7}	Aggregate
7	853	418	2.04	6.2×10^{7}	Aggregate
8	829	515	1.61	9.16×10^{7}	Aggregate
9	718	382	1.88	4×10^{7}	Aggregate
10	390	193	2.02	4.1×10^{6}	Aggregate
11	273	72	3.80	1.08×10^{6}	Isolated
12	243	138	1.77	1.3×10^{6}	Isolated
13	222	72	3.09	864000	Isolated
14	66	66	1.00	216000	Isolated

Supplementary Table 2: Results of the quantitative textural analysis performed on the 3D images of sample MTL-A, showing the size of the long and short axes and the volume of both nanolite aggregates and individual nanolites where they are isolated.

Label	Long axis (nm)	Short axis (nm)	Aspect ratio	Volume (nm^3)	Type
1	3310	2304	1.44	1.64×10^9	Aggregate
2	506	336	1.51	2.39×10^7	Aggregate
3	361	167	2.16	3.6×10^6	Aggregate
4	286	162	1.76	2.64×10^6	Isolated
5	212	114	1.87	1.27×10^6	Isolated
6	130	88	1.48	386904	Isolated
7	126	87	1.45	367696	Isolated
8	61	41	1.48	30184	Isolated
9	52	23	2.28	13720	Isolated
10	32	15	2.07	5488	Isolated
11	15	15	1.00	2744	Isolated

Supplementary Table 3: Results of the quantitative textural analysis performed on the 3D images of sample MTL-B.

**Sample**	**Volume[a] (nm³)**	**Area[b] (nm²)**	**Long axis[c] (nm)**	**Short axis[d] (nm)**	**Volume[e] (nm³)**	**Volume[f] (nm³)**
MTL-A						
3D	1.05×10^{10}					
Slice 1		2.98×10^{6}	2000	1200	5.95×10^{9}	3.57×10^{9}
Slice 2		2.06×10^{6}	1800	900	3.71×10^{9}	1.85×10^{9}
Slice 3		9.25×10^{5}	1500	600	1.39×10^{9}	5.55×10^{8}
Slice 4		7.25×10^{5}	600	300	4.35×10^{8}	2.17×10^{8}
MTL-B						
3D	1.64×10^{9}					
Slice 1		3.85×10^{5}	600	500	2.31×10^{8}	1.93×10^{8}
Slice 2		6.97×10^{5}	1400	400	9.75×10^{8}	2.79×10^{8}
Slice 3		9.75×10^{5}	1000	500	9.75×10^{8}	4.87×10^{8}
Slice 4		1.99×10^{5}	400	300	7.95×10^{7}	5.96×10^{7}

[a] Nanolite aggregate volume estimated in 3D using the reconstructed volume obtained from X-ray ptychography.

[b] Nanolite aggregate area estimated in 2D using an orthoslice from the 3D reconstructed volume.

[c] Long axis of the nanolite aggregate, measured in 2D using the orthoslice.

[d] Short axis of the nanolite aggregate, measured in 2D using the orthoslice.

[e] Nanolite aggregate volume estimated using a correction considering the long axis of the aggregate measured in 2D.

[f] Nanolite aggregate volume estimated using a correction considering the short axis of the aggregate measured in 2D.

Supplementary Table 4: Estimated volume of the nanolite aggregate comparing 3D and 2D characterisation methods. The estimated volume of the nanolite aggregate in 3D uses the reconstructed volume acquired using X-ray ptychography. The volume of the nanolite aggregate is estimated in 2D using a single orthoslice from the 3D reconstructed volume. The 2D estimate considers a correction applied using the long and short axis of the aggregate to convert the measured area into an estimate of the volume. The results from 4 different orthoslices are considered for both samples MTL-A and MTL-B, to compare the volume estimated using sections in different orientations. The volume estimated from the 2D image strongly depends on the cross-section of the aggregate, with some slices providing a more representative estimate of the 3D volume. All 2D textural measurements were performed using ImageJ[1].

	Giordano et al. (2008)	Langhammer et al. (2022)
**Basaltic melt composition**	6.1×10^1	1.3×10^1
**Evolved melt composition**	3.2×10^2	3.5×10^1
**Vona et al. (2011)**	1.2×10^4	1.4×10^3
**Mader et al. (2013)**	1.4×10^3	1.5×10^2

Supplementary Table 5: Results of the magma viscosity calculations (in Pa s). The Giordano et al.[4] model was first used to calculate the melt viscosity. The basaltic melt composition is the average melt inclusion composition of the MTL eruption[2], whilst the evolved melt composition refers to the trachy-andesitic composition of the glass which surrounds nanolites[3]. These estimates of melt viscosity were then used in subsequent calculations using the Vona et al.[5] model and the Krieger and Dougherty[6] equation to estimate the magma viscosity (melt + crystals). The calculations using the Krieger and Dougherty[6] equation follow the approach of Mader et al.[7]. All calculations were performed using a temperature of 1080 ˚C and H_2O content of 2 wt.%, consistent with the pre-eruptive conditions defined for the MTL eruption from the results of petrological analysis[2]. Here, the calculations of the melt viscosity using the Giordano et al.[4] model are also compared with the results using the model of Langhammer et al[8].

Supplementary Methods

Calculations of magma viscosity

In the main text we present the results of the calculations for the melt viscosity using the Giordano et al.[4] model. For completeness, here we also compare these results with the calculation of melt viscosity using the recent Langhammer et al.[8] model, based on artificial neural networks. The Langhammer et al.[8] model predicts a lower crystal-free melt viscosity of 1.3 x 10^1 Pa s, compared to the estimate of 6.1 x 10^1 Pa s calculated using the Giordano et al.[4] model (Supplementary Table 5). Considering also the Langhammer et al.[8] model uncertainty, the melt viscosity calculated using this model ranges between 9 and 1.9 x 10^1 Pa s.

Using the Langhammer et al.[8] model to calculate the viscosity of the evolved melt composition provides a value of 3.5 x 10^1 Pa s, lower than the viscosity estimated by the Giordano et al.[4] model of 3.2 x 10^2 Pa s. Considering also the Langhammer et al.[8] model uncertainty, the estimated viscosity of the evolved melt composition using this model ranges between 2.4 x 10^1 and 5.2 x 10^1 Pa s.

Incorporating the estimate of melt viscosity from the Langhammer et al.[8] model in the equation of Costa et al.[9] to estimate the magma viscosity (melt + crystals), provides a value of 1.4 x 10^3 Pa s using the Vona et al.[5] parameters. This estimate of the magma viscosity is lower than that estimated using the Giordano et al.[4] model of 1.2 x 10^4 Pa s.

However, comparison of experimental datasets investigating the relative increase in magma viscosity attributed to crystallisation indicates that the estimated viscosity increase can vary considerably for the same value of crystal fraction[9, 10], particularly at high crystal fractions. This variation may arise from the uncertainties associated with the calculated proportions of melt and crystals in the experiments and the experimental conditions[9]. Variations in the fitting parameters for the Costa et al.[9] equation can lead to uncertainties in the relative viscosity increase resulting from crystallisation. Here, we use the values for these parameters derived from the rheological experiments of Vona et al.[5] in our calculations, performed on crystal-bearing basaltic

compositions. Although this variation may lead to uncertainties in our estimates of the magma viscosity, we use several viscosity models in our calculations and the experimental data of Vona et al.[5] to estimate the viscosity of the nanolite-bearing MTL composition. Furthermore, there is less variation in the relative viscosity increase estimated for different values of these parameters at the lower crystal fractions measured in this study (0.27 ± 0.02).

Finally, we also used the Krieger and Dougherty[6] equation for particle-bearing suspensions, and the approach presented in Mader et al.[7] to estimate the magma viscosity, incorporating our 3D data on aspect ratio (r_p). Using the average r_p of 2 (Supplementary Tables 2-3), the calculated viscosity is 1.4×10^3 Pa s.

Supplementary References

1. Abràmoff, M.D., Magalhães, P.J. & Ram, S.J. Image processing with ImageJ. *Biophoton. Int.* **11,** 36-42 (2004).
2. Bamber, E.C. et al. Pre-and syn-eruptive conditions of a basaltic Plinian eruption at Masaya Volcano, Nicaragua. *J. Volcanol. Geotherm. Res.* **392,** 106761 (2020).
3. Bamber, E.C. et al. Basaltic Plinian eruptions at Las Sierras-Masaya volcano driven by cool storage of crystal-rich magmas. *Commun. Earth Environ.* **3,** 253 (2022).
4. Giordano, D. Russell, J.K. & Dingwell, D.B. Viscosity of magmatic liquids: A model. *Earth Planet. Sci. Lett.* **271,** 123-134 (2008).
5. Vona, A., Romano, C., Dingwell, D.B. & Giordano, D. The rheology of crystal-bearing basaltic magmas from Stromboli and Etna. *Geochim. Cosmochim. Acta* **75,** 3214-3236 (2011).
6. Krieger, I.M. and Dougherty, T.J. A mechanism for non-Newtonian flow in suspensions of rigid spheres. *Trans. Soc. Rheol.* **3**, 137-152 (1959).
7. Mader, H.M., Llewellin, E.W. & Mueller, S.P. The rheology of two-phase magmas: A review and analysis. *J. Volcanol. Geotherm. Res.* **257,** 135-158 (2013).
8. Langhammer, D., Di Genova, D. & Steinle-Neumann, G. Modeling viscosity of volcanic melts with artificial neural networks. *Geochem. Geophys. Geosyst.* **23,** e2022GC010673 (2022).
9. Costa, A., Caricchi, L. & Bagdassarov, N. A model for the rheology of particle-bearing suspensions and partially molten rocks. *Geochem. Geophys. Geosyst.* **10,** 3 (2009).
10. Mueller, S., Llewellin, E.W. & Mader, H.M. The rheology of suspensions of solid particles. *Proc. R. Soc. A*, **466**, 1201-1228 (2010).
